# The complete chloroplast genome and phylogenetic analysis of *Urceola huaitingii* (Chun & Tsiang) D. J. Middleton 1994 (Apocynaceae)

**DOI:** 10.1080/23802359.2026.2710930

**Published:** 2026-08-11

**Authors:** Xianglan Liang, Guoan Shen, Lichai Yuan, Jiahua Chen, Song Guo, Chang Liu

**Affiliations:** a School of Chemistry and Chemical Engineering, Guangdong Pharmaceutical University, Guangzhou, PR China; b Institute of Medicinal Plant Development, Chinese Academy of Medical Sciences & Peking Union Medical College, Beijing, PR China; c College of Smart Agriculture, Guangxi Science & Technology Normal University, Laibin, PR China

**Keywords:** *Urceola huaitingii*, chloroplast genome, phylogenetic analysis, medicinal plant

## Abstract

*Urceola huaitingii* (Chun & Tsiang) D. J. Middleton 1994, a member of the Apocynaceae family, is widely distributed across southern and southwestern China and has been traditionally used in folk medicine to treat hemiplegia and paralysis. In this study, we report the first complete chloroplast genome of *U. huaitingii* and perform phylogenetic analysis with 30 related species within the Apocynaceae. The chloroplast genome of *U. huaitingii* is 155,182 bp in length and has a GC content of 38.11%. It displays a typical quadripartite structure, consisting of a large single-copy (LSC) region of 85,254 bp, a small single-copy (SSC) region of 18,242 bp, and two inverted repeat (IR) regions of 25,843 bp each. A total of 111 unique genes were annotated, including 77 protein-coding genes, 30 transfer RNA (tRNA) genes, and 4 ribosomal RNA (rRNA) genes. Phylogenetic analysis revealed that *U. huaitingii* is closely related to the genera *Aganosma*, *Trachelospermum*, and *Amalocalyx*. This study provides the first chloroplast genomic resource for the genus *Urceola*, laying a foundation for future investigations into its evolutionary relationships. It also contributes to future molecular and phylogenetic studies within the Apocynaceae family.

## Introduction

*Urceola huaitingii* (Chun & Tsiang) D. J. Middleton 1994, also known as *Parabarium huaitingii* (Middleton 1994), is a perennial plant in the genus *Urceola* of the family Apocynaceae. It is widely distributed across the southern and southwestern regions of China. As a traditional folk medicinal plant, *U. huaitingii* is used to dispel wind, activate the collaterals, and strengthen muscles and bones. It has been applied in the treatment of rheumatic pain (Lei et al. 2011), lumbar strain, paralysis, and other related conditions. Phytochemical investigations have revealed that *U. huaitingii*, particularly its stems, is rich in proanthocyanidins and (−)-epicatechin, compounds known for their potential antitumor (Yu et al. 2016) and anti-myocardial infarction activities (Tang et al. 2011), along with other significant pharmacological activities.

To date, research on the genus *Urceola* has mainly focused on its chemical constituents and biological activities. Species identification and phylogenetic relationships within the genus remain controversial, but molecular data for *U. huaitingii* are particularly scarce. No chloroplast genome data have been reported for this genus, limiting phylogenetic and evolutionary studies of *Urceola* and its related taxa. In this study, we sequenced and assembled the complete chloroplast genome of *U. huaitingii*, providing a foundational genomic resource for future phylogeographic, taxonomic, and population genetic studies of this species.

## Materials and methods

**Figure 1.**
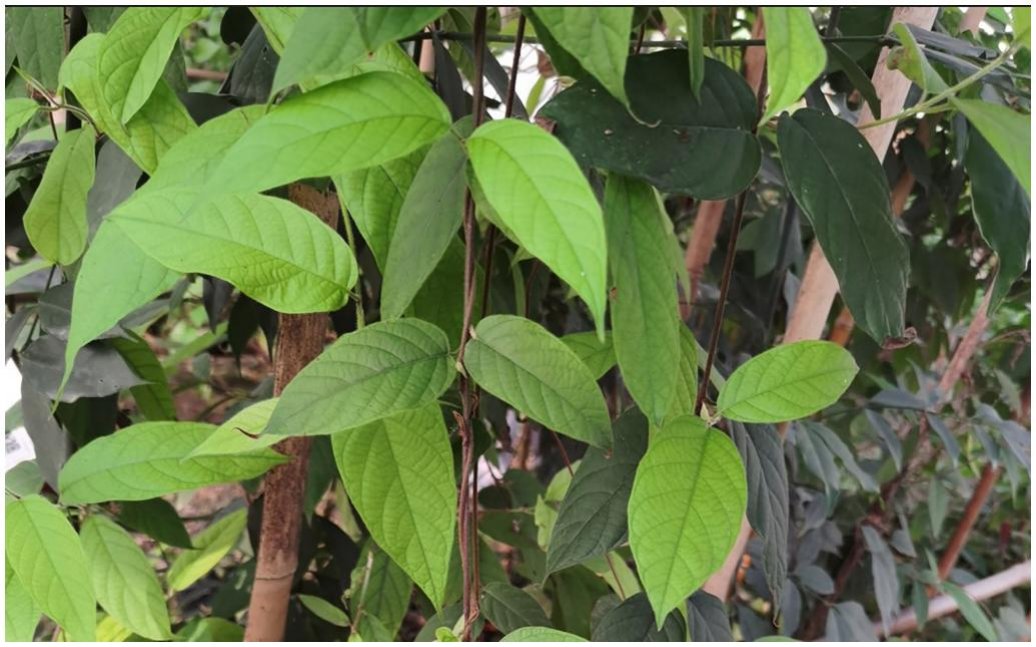
Leaf of *U. huaitingii*, photographed by Jiahua Chen in Jinxiu Yao Autonomous County, Guangxi, China. *U. huaitingii* is a liana with leaves borne at branch apices. The leaf blade is elliptic to oblong-elliptic, with slightly revolute margins, an acute to shortly acuminate apex, and a narrow to broadly cuneate base. Both surfaces are pubescent, with dense pubescence along the abaxial veins. The adaxial surface is dark green, and the abaxial surface is pale green.

Fresh and healthy leaves of *U. huaitingii* were collected from Jinxiu Yao Autonomous County, Guangxi Province, China (24°10′39.4″N, 110°0′31.5″E) (Figure 1). The plant specimen was identified by Mr. Zhaosheng Pang, and a voucher specimen (No. JXHC011) was deposited at the College of Biology and Food Engineering of Guangxi Science & Technology University (Contact: Song Guo, guosong0804@163.com).

Total genomic DNA was extracted using a modified CTAB method. High-quality DNA was then sequenced on the Illumina NovaSeq 2500 platform (Illumina, San Diego, CA, USA), generating 150 bp paired-end reads with a total data volume of approximately 9.95 GB. The raw reads were subjected to quality control using fastp v0.23.2 (Chen 2023) to remove low-quality reads and adapter sequences. The filtered reads were assembled into a complete chloroplast genome using GetOrganelle v1.7.7.0 (Jin et al. 2020) with default parameters. The resulting circular genome was visualized using Bandage v0.8.1 (Jin et al. 2020). To assess sequencing depth and genome coverage, clean reads were mapped back to the assembled chloroplast genome using BWA v0.7.17 (Li and Durbin 2009). The resulting alignments were processed using Samtools v1.13 (Danecek et al. 2021), and sequencing depth statistics were calculated from the BAM files. Genome annotation was performed using CPGAVAS2 (Shi et al. 2019), with *Chonemorpha eriostylis* (NC_072067.1) as the reference. The annotation of tRNA genes was further verified using tRNAscan-SE v2.0 (Chan et al. 2021). Manual corrections of start and stop codons, as well as intron/exon boundaries, were performed using Geneious Prime 2025, based on the chloroplast genome of *C. eriostylis*. The chloroplast genome map, along with the cis-splicing genes and the trans-splicing gene (*rps*12), was generated using CPGView (Liu et al. 2023). To further investigate structural variation among closely related species, IRScope (https://irscope.shinyapps.io/irapp/) was used to analyze the expansion and contraction of inverted repeat (IR) regions. The complete chloroplast genome sequence and its annotations were submitted to GenBank database (Accession number: PV608489).

For phylogenetic analysis, chloroplast genome sequences from 30 additional species within Apocynaceae were retrieved from the National Center for Biotechnology Information (NCBI). Based on a previously reported approach (Kim et al. 2023), 65 shared protein-coding sequences (CDSs) were extracted across all species using PhyloSuite (Xiang et al. 2023) for downstream analysis. Two species from the family Phrymaceae, *Mimulus ringens* (NC_068043.1) and *Diplacus grandiflorus* (NC_068025.1), were used as outgroups. Multiple sequence alignments were generated utilizing MAFFT v7.490 (Katoh et al. 2002). Maximum likelihood (ML) trees were constructed using IQ-TREE v2.3.3(Lanfear et al. 2020), under the best-fit model GTR+F + R3, and node support was assessed with 1000 bootstrap replicates. The resulting phylogenetic tree was visualized using the Interactive Tree of Life (iTOL) online tool (https://itol.embl.de/) (Letunic and Bork 2024).

## Results

The complete chloroplast genome sequence of *U. huaitingii* was submitted to the GenBank database under accession number PV608489. The raw sequencing reads have been deposited in the GenBank Sequence Read Archive (SRA) under accession number SRR33650030. The sequencing exhibited high quality, with an average depth of coverage of 847.33 x. No uncovered regions were observed, indicating a high level of assembly completeness and reliability (Figure S1).

**Figure 2.**
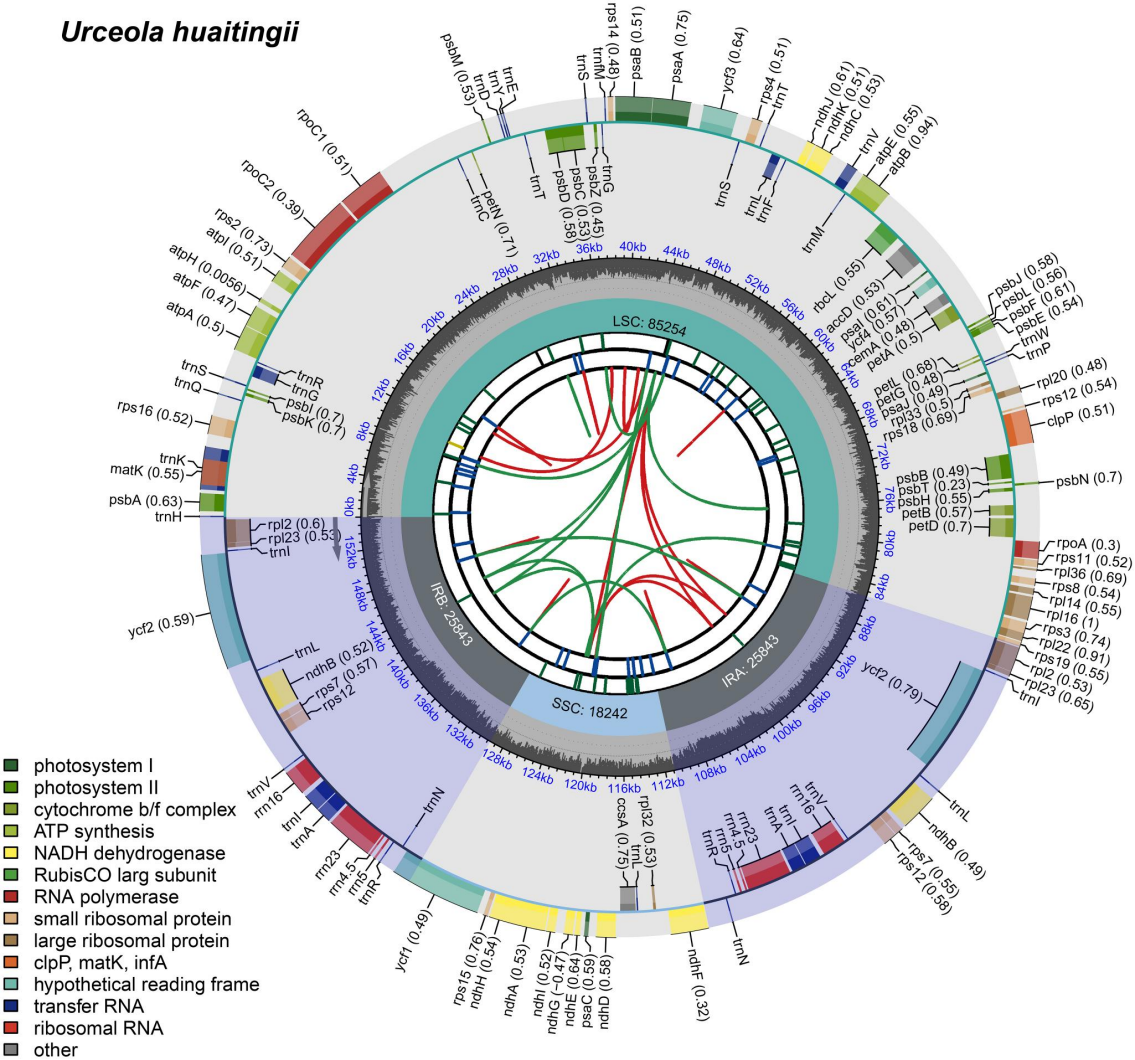
Schematic map illustrating the overall features of the chloroplast genome of *U. huaitingii*. The map comprises six concentric tracks by default. From the center outward: the first track displays dispersed repeats; the second shows long tandem repeats as short blue bars; the third indicates short tandem repeats (microsatellites) as colored bars. The fourth track marks the structural regions of the genome, including SSC, LSC, IRa, and IRb. The fifth track plots the GC content across the genome. The sixth track presents annotated genes, with optional codon usage bias shown in parentheses after gene names. Genes are color-coded based on functional categories, as indicated in the legend at the bottom left. Genes transcribed on the inner and outer circles are oriented clockwise and counterclockwise, respectively.

The chloroplast genome of *U. huaitingii* is 155,182 bp in length and has an overall GC content of 38.11%. It exhibits the typical quadripartite structure, consisting of a large single-copy (LSC) region of 85,254 bp, a small single-copy (SSC) region of 18,242 bp, and a pair of inverted repeat (IR) regions, each 25,843 bp in length (Figure 2). The GC content varies across different regions: 36.26% in the LSC, 31.90% in the SSC, and 43.35% in the IRs. A total of 128 genes (111 unique genes) were annotated in the chloroplast genome, including 83 protein-coding genes (77 are unique), 37 tRNA genes (30 are unique), and 8 rRNA genes (4 are unique). Among the protein-coding genes, 8 were identified as cis-splicing genes, each containing a single intron (Figure S2). Additionally, two genes (*ycf*3 and *clp*P) contain two introns. The *rps*12 gene was characterized as a trans-splicing gene (Figure S3). Furthermore, IR boundary analysis revealed a relatively conserved structure among species within the same family (Figure S4).

**Figure 3.**
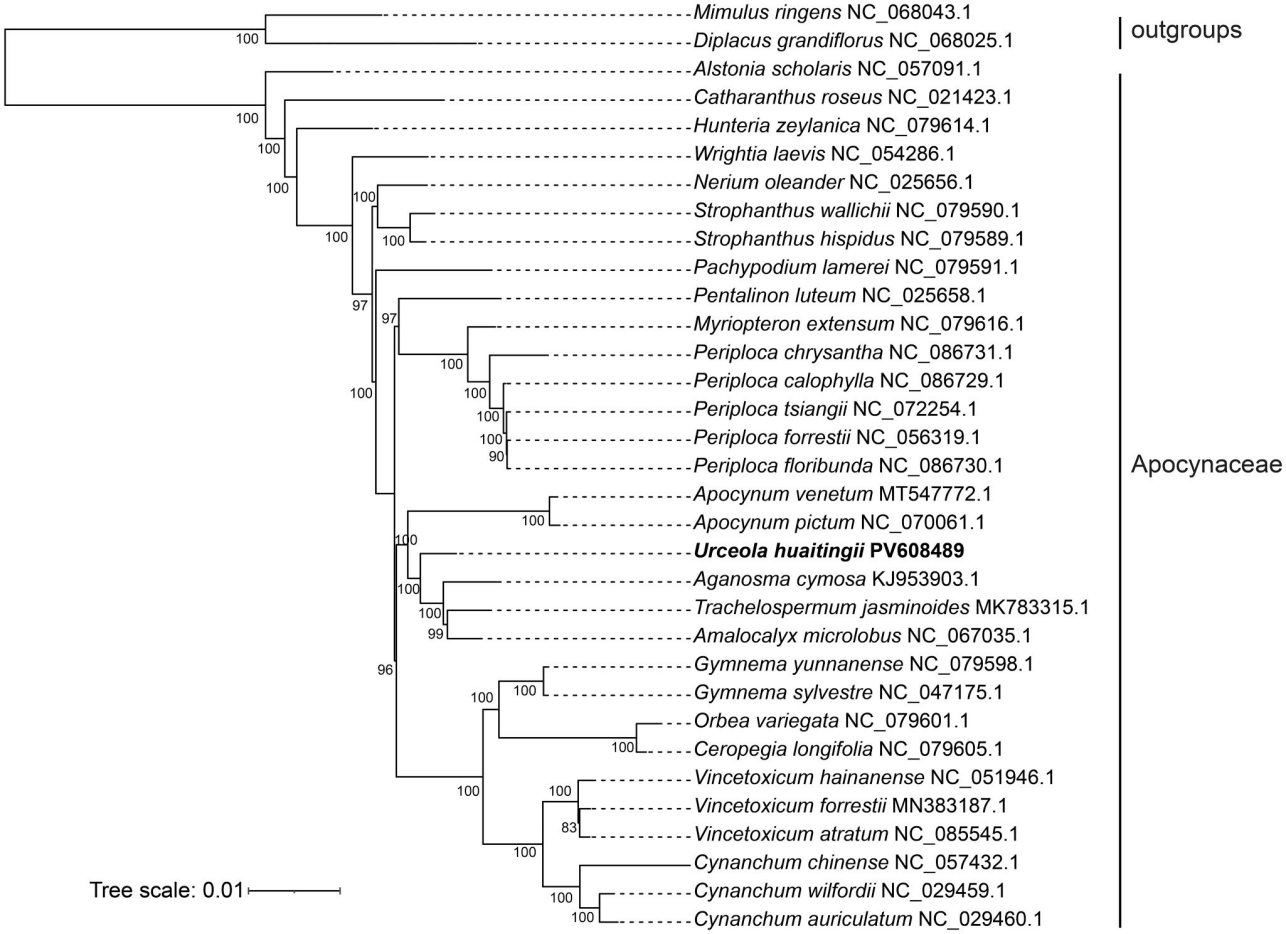
Phylogenetic tree of 30 Apocynaceae species inferred using the ML method based on 65 shared CDSs. *M. ringens* (NC_068043.1) and *D. grandifloras* (NC_068025.1) was selected as outgroup. Numbers at each node represent the bootstrap values. The position of *U. huaitingii* is marked in bold. The sequences used for constructing the phylogenetic tree are as follows: *Trachelospermum jasminoides* (MK783315.1) (Wang et al. 2019), *Amalocalyx microlobus* (NC_067035.1), *Periploca calophylla* (NC_086729.1), *Periploca tsiangii* (NC_072254.1), *Periploca floribunda* (NC_086730.1), *Periploca forrestii* (NC_056319.1) (Long et al. 2022), *Periploca chrysantha* (NC_086731.1), *Apocynum venetum* (MT547772.1) (Chang et al. 2020), *Strophanthus hispidus* (NC_079589.1), *Strophanthus wallichii* (NC_079590.1), *Pentalinon luteum* (NC_025658.1), *Pachypodium lamerei* (NC_079591.1), *Hunteria zeylanica* (NC_079614.1), *Aganosma cymose* (KJ953903.1), *Apocynum pictum* (NC_070061.1), *Nerium oleander* (NC_025656.1), Vincetoxicum hainanense (NC_051946.1) (Xiong et al. 2019), *Vincetoxicum forrestii* (MN383187.1), *Vincetoxicum atratum* (NC_085545.1) (Ren et al. 2025), *Orbea variegata* (NC_079601.1), *Catharanthus roseus* (NC_021423.1) (Ku et al. 2013), *Gymnema sylvestre* (NC_047175.1), *Gymnema yunnanense* (NC_079598.1), *Myriopteron extensum* (NC_079616.1), *Wrightia laevis* (NC_054286.1) (Li et al. 2020), *Ceropegia longifolia* (NC_079605.1), *Ceropegia nilotica* (NC_079603.1), *Cynanchum chinense* (NC_057432.1), *Cynanchum auriculatum* (NC_029460.1) (Jang et al. 2016), *Cynanchum wilfordii* (NC_029459.1) (Park et al. 2016), *M. ringens* (NC_068043.1), *D. grandifloras* (NC_068025.1).

Phylogenetic analysis based on 65 shared CDSs from 30 species across 19 genera within the Apocynaceae family revealed the phylogenetic position of *U. huaitingii* (Figure 3). The results indicated that *U. huaitingii* is closely related to species in the genera *Aganosma*, *Trachelospermum*, and *Amalocalyx*, with strong bootstrap support.

## Discussion and conclusion

The chloroplast genome is known for its relatively conserved structural organization. The chloroplast genome of *U. huaitingii* shared similar features with other genera in the Apocynaceae family, particularly in genome structure and composition. Previous studies showed that the GC content in this family ranged from 37% to 39% (Long et al. 2022; Zhang et al. 2024), and the genome size varied from approximately 150–165 kb (Ku et al. 2013; Li et al. 2020). Cis-splicing genes were also commonly identified among species within the family (Kim et al. 2023; Zhang et al. 2023). In addition, the number of genes generally ranged from 128 to 142 (Zhang et al. 2024; Abdullah et al. 2025).

Genes located at the IR boundaries were mainly *rps19*, *ycf1*, *ndhF*, and *rpl2* (Abba et al. 2021), further supporting the conserved nature of chloroplast genome organization in the family. Nevertheless, differences in gene length and IR boundary positions were observed among species. For example, the length of *ycf1* in *Apocynum venetum* differed by 108 bp compared with that in *U. huaitingii*, whereas *P. floribunda* exhibits the same *ycf1* length as *U. huaitingii*. Variations in the distribution and length of *ycf1* at the SSC/IRb boundary suggested the occurrence of IR contraction and expansion events, which may have contributed to chloroplast genome evolution within the family.

Phylogenetic analysis elucidated the phylogenetic position of *U. huaitingii* within the family, showing that it had a close genetic relationship with *Aganosma*, *Trachelospermum*, and *Amalocalyx* (Figure 3). Most nodes in the phylogenetic tree received high bootstrap support values, indicating the robustness and reliability of the inferred relationships. The phylogenetic tree displayed consistent topology with the previous reports (Chang et al. 2020; Long et al. 2022; Liu et al. 2023).

In this study, the complete chloroplast genome of *U. huaitingii* was assembled and reported for the first time, representing the first such record for the genus. This provides a valuable resource for species classification and phylogenetic studies within *Urceola* and the Apocynaceae family. These findings also offer important genomic data for molecular identification and evolutionary analysis of the Apocynaceae family. Further studies with broader taxon sampling and multi-genome data will help deepen our understanding of the evolutionary history of *Urceola*.

## Supplementary Material

Supplemental Material

Supplemental Material

Supplemental Material

Supplemental Material

## Data Availability

The genome sequence data that support the findings of this study are publicly available from the NCBI GenBank database under accession number PV608489. The associated BioProject, BioSample, and SRA accession numbers are PRJNA1265672, SAMN48632344, and SRR33650030, respectively.
